# First person – Anam Naseer

**DOI:** 10.1242/dmm.052654

**Published:** 2025-11-10

## Abstract

First Person is a series of interviews with the first authors of a selection of papers published in Disease Models & Mechanisms, helping researchers promote themselves alongside their papers. Anam Naseer is first author on ‘ Knockdown of mitochondrial sirtuin *sir-2.2* reduces alpha-synuclein clearance and impairs energy homeostasis in a model of ageing’, published in DMM. Anam is a research associate in the lab of Dr Aamir Nazir at CSIR-Central Drug Research Institute, Lucknow, India, investigating the genetics and epigenetics of ageing biology and age-associated neurodegeneration.


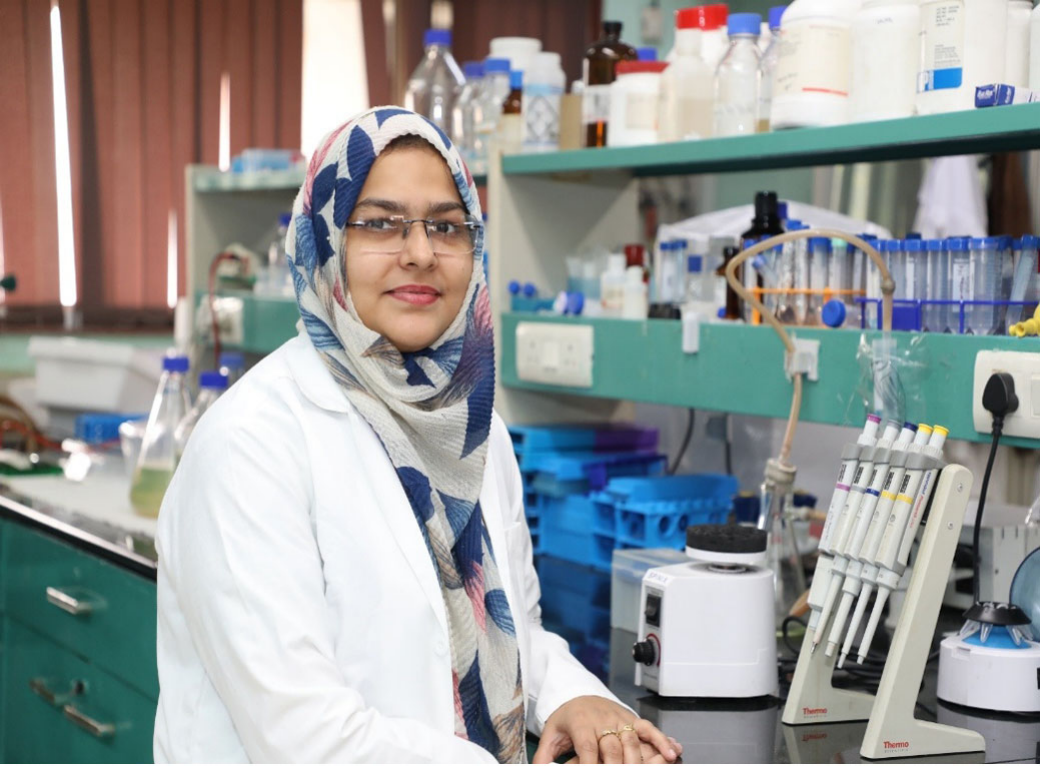


**Anam Naseer**

**Who or what inspired you to become a scientist?**

Since I was a kid, nature and its intricacies have always fascinated me and lured me to choose science as my major. During my studies, I gained a wider knowledge regarding the fundamentals of biology and related techniques. To understand nature closely, I started working on the complex interplay between ageing biology and sirtuins, employing a powerful tool, *Caenorhabditis elegans*, as model during my PhD research work. My interest in practical applications of science led me to pursue my career in research and do some exciting science.

**What is the main question or challenge in disease biology you are addressing in this paper? How did you go about investigating your question or challenge?**

Ageing and associated neurodegeneration pose great challenges for designing potential drugs for curing the most prominent nondegenerative disorders such as Alzheimer's disease and Parkinson's disease. Also, the class 3 HDACs, called sirtuins, hold therapeutic potential since they are associated with longevity. Thus, to explore the potential of these proteins, in the current study, we investigated the mechanistic aspect of mitochondrial sirtuins in modulating age-associated neurodegeneration, specifically Parkinson's disease. With the help of RNA interference-based gene knockdown, we silenced the mitochondrial sirtuin *sir-2.2* in a well-established model system, *C. elegans*. Because of its crucial role in regulating oxidative stress and mitochondrial quality control, we studied the effect of this knockdown in modulating Parkinson's disease pathology. This work will aid in providing therapeutic insights into the metabolic regulation of ageing and neurodegeneration.

**How would you explain the main findings of your paper to non-scientific family and friends?**

In simple terms, I can say that my work highlights the significance of proteins that help in restoring energy balance, clean up junk material and, most importantly, help in regulating neurobehavioral response. Also, these proteins form an integral part of the powerhouse of the cell and impart better immune competence.

**What are the potential implications of these results for disease biology and the possible impact on patients?**

Discoveries of this study highlight the critical role of the mitochondrial sirtuin SIR-2.2 in neuroprotection and mitochondrial homeostasis. These findings have helped in advancing our understanding of the mechanistic aspects of age-associated neurodegenerative diseases. Being pathophysiologically active, sirtuins offer great potential as therapeutic targets. Mitochondrial sirtuins interact with mTOR regulators and inhibit tumour progression, and also sense nutrient deficiency via NAD^+^, thus activating the AMPK pathway to restore balance. Thus, these proteins provide promising molecules for designing therapeutics. In-depth study of these proteins will aid in leveraging these pathways for successful translational applications.

… my work highlights the significance of proteins that help in restoring energy balance, clean up junk material and, most importantly, help in regulating neurobehavioral response

**Figure:**
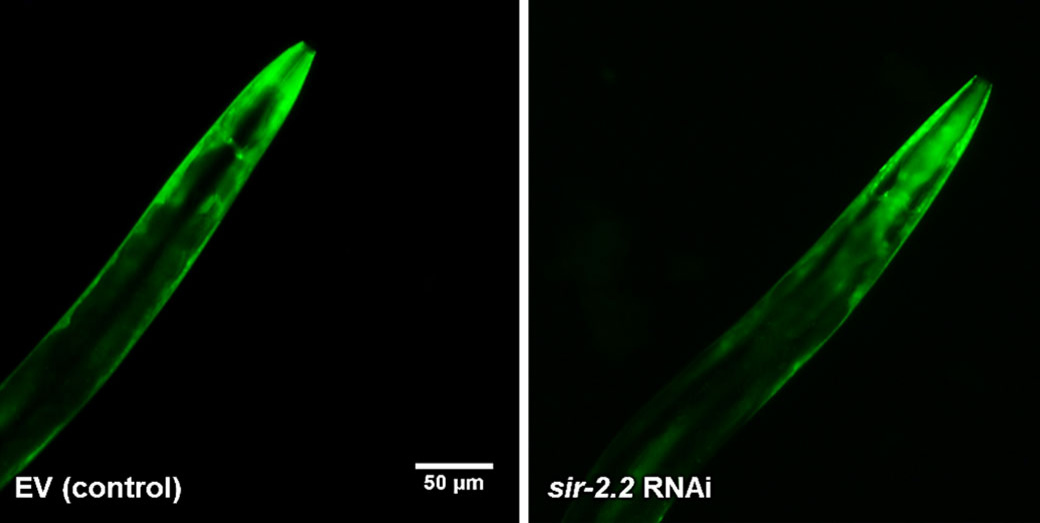
***sir-2.2* knockdown increases alpha-synuclein aggregation (green fluorescence) and aggravates Parkinson's disease pathology.** EV, empty vector; RNAi, RNA interference.

**Why did you choose DMM for your paper?**

Owing to its research-centric approach, DMM excels as one of the best journals in the field of disease biology. Because of its multi-tier screening process and hassle-free communication procedure, DMM became our first choice to communicate our work. Since the present research is focused on age-associated disease regulation, it aligns well with the aims of the journal.

**Given your current role, what challenges do you face and what changes could improve the professional lives of other scientists in this role?**

Currently, I am working as a research associate, and my PhD life was a big rollercoaster since I had to play the dual role of mother and researcher. During this journey, my major task challenge was to balance my work along with my hyperactive toddler. I feel that research-wise collaboration between mechanistic aspects of disease biology and translational research could help us to understand Parkinson's disease. Thus, to address this concern, there is a need for analysing the disease in a practical perspective and designing therapeutics that would be translational in nature and help in improving the disease pathology. For implementing this idea, there is a requirement for good funding and better research facilities that will make the work easier and quicker.

**What's next for you?**

In the coming years, I envision becoming an independent researcher. I aspire to become a great scientist, leading high-quality research with a meaningful impact on science, reducing the disease burden and working towards betterment of the society.

**Tell us something interesting about yourself that wouldn't be on your CV**

Besides experimenting in the lab, I like to try new recipes and I enjoy cooking. In addition to that, I am a melophile, who relishes music.
